# Two co-dependent routes lead to high-level MRSA

**DOI:** 10.1126/science.adn1369

**Published:** 2024-10-31

**Authors:** Abimbola Feyisara Adedeji-Olulana, Katarzyna Wacnik, Lucia Lafage, Laia Pasquina-Lemonche, Mariana Tinajero-Trejo, Joshua A. F. Sutton, Bohdan Bilyk, Sophie E. Irving, Callum J. Portman Ross, Oliver J. Meacock, Sam A. Randerson, Ewan Beattie, David S. Owen, James Florence, William M. Durham, David P. Hornby, Rebecca M. Corrigan, Jeffrey Green, Jamie K. Hobbs, Simon J. Foster

**Affiliations:** 1 Department of Physics and Astronomy, University of Sheffield, Sheffield, UK; 2 School of Biosciences, University of Sheffield, Sheffield, UK; 3 The Florey Institute, University of Sheffield, Sheffield, UK; 4 School of Medicine, University College Dublin, Dublin, Ireland

## Abstract

Methicillin resistant *S. aureus* (MRSA) is of major clinical concern, in which acquisition of *mecA*, encoding the cell wall peptidoglycan biosynthesis component Penicillin Binding Protein 2a (PBP2a), confers resistance to β-lactam antibiotics. In the presence of antibiotics we show that MRSA adopts an alternative cell division mode, with altered peptidoglycan architecture at the division septum. PBP2a can replace the transpeptidase activity of the endogenous and essential PBP2, but not that of PBP1, which is responsible for the distinctive native septal peptidoglycan architecture. Successful division without PBP1 activity, requires the alternative division mode and is enabled by several possible chromosomal, potentiator (*pot*) mutations. MRSA resensitizing agents differentially interfere with the two co-dependent mechanisms required for high-level antibiotic resistance, providing opportunities for new interventions.

## Introduction

Antibiotics are at the heart of modern medicine, but their efficacy is increasingly challenged by the spread of antimicrobial resistance (AMR) (1). MRSA is a so-called AMR “superbug”, that causes over 120,000 deaths per annum (2). Methicillin was introduced to circumvent clinical β-lactamase-mediated resistance, but soon became compromised due to the spread of MRSA (3). Resistance in MRSA is primarily based on the acquisition of the *mecA* gene encoding a novel PBP, named PBP2a, characterised by its low affinity for a broad range of β-lactams (3, 4). The *mecA* gene is carried on a mobile genetic element, the staphylococcal cassette chromosome (SCC*mec*) (3). SCC*mec* elements are classified into several types, including I, II, and III, which are primarily hospital-associated clones, and types IV and V often identified in community-associated MRSA (3).

PBPs are enzymes that carry out the final stages of assembly of bacterial cell wall peptidoglycan (PG). Cell wall PG is essential for viability of most bacteria and forms a single macromolecule around the cell (the sacculus), made of glycan strands and cross-linked via peptide side-chains (5). High resolution Atomic Force Microscopy (AFM) has recently revealed *S. aureus* PG to be a porous, heterogeneous hydrogel (6). Its mature surface is an open, disordered mesh with pores that penetrate deep into the wall, whereas the inner surface, where PG is synthesised, is a much denser mesh (6). Another feature of the PG is an outer architecture of concentric rings consisting of long glycan strands that is revealed upon cell scission and is characteristic of the newly exposed septum (6).

*S. aureus* has four endogenous PBPs of which only PBP1 and 2 are essential for PG synthesis, being able to carry out all the transpeptidase (linking side-chains) functions necessary for cell growth and division (7, 8, 9). PBP1 has multiple roles in cell division, by acting as a coordinator, through interactions with PG and divisome protein partners, and by providing the transpeptidase activity that is thought to be required for the characteristic ring architecture in septal PG (6, 7, 8).

PBP2a is a non-native enzyme in MRSA, acquired from an environmental source, so how it facilitates high-level antibiotic resistance by replacing the transpeptidase activity of endogenous PBPs is intriguing. PBP2a requires the transglycosylase activity of PBP2 to mediate resistance and the two proteins interact, thus demonstrating their functional cooperativity (10). PBP2a can maintain transpeptidase activity with a closed active site conformation, thus resisting β-lactam binding while interaction with a second PG substrate molecule at an allosteric site leads to a conformational change that opens the active site for catalysis (11).

An interesting feature of many clinical MRSA isolates is that they exhibit heterogeneous resistance, whereby only a very small proportion (<10^-4^) of the population are high-level resistant (>50 μg ml^-1^ methicillin) (12). Antibiotics can induce the conversion of the population to homogeneous high-level resistance, that does not revert in the absence of antibiotics. Chromosomal mutations that lead to the conversion to homogeneous resistance, mostly map to genes responsible for the regulation of aspects of cellular physiology and not PBP2a function directly (13). We have named these genes “potentiators” (*pot*), to differentiate them from auxiliary genes (*aux*), in which mutation leads to decreased resistance (13). We have recently carried out a directed evolution study that provides matched strains enabling the exploration of MRSA resistance mechanisms (14). Development of high-level MRSA is a two-step process whereby the presence of *mecA* is essential but in itself only results in a modest increase in minimum inhibitory concentration (MIC) (low-level MRSA). Acquisition of missense mutations in genes encoding RNA polymerase subunits (*rpoB* or *rpoC*), so-called rpo* mutations, potentiate a step-change in resistance levels (high-level MRSA), both in the clinical environment and under laboratory conditions (13, 14).

### Cell wall architecture of MRSA

**Fig. 1:**
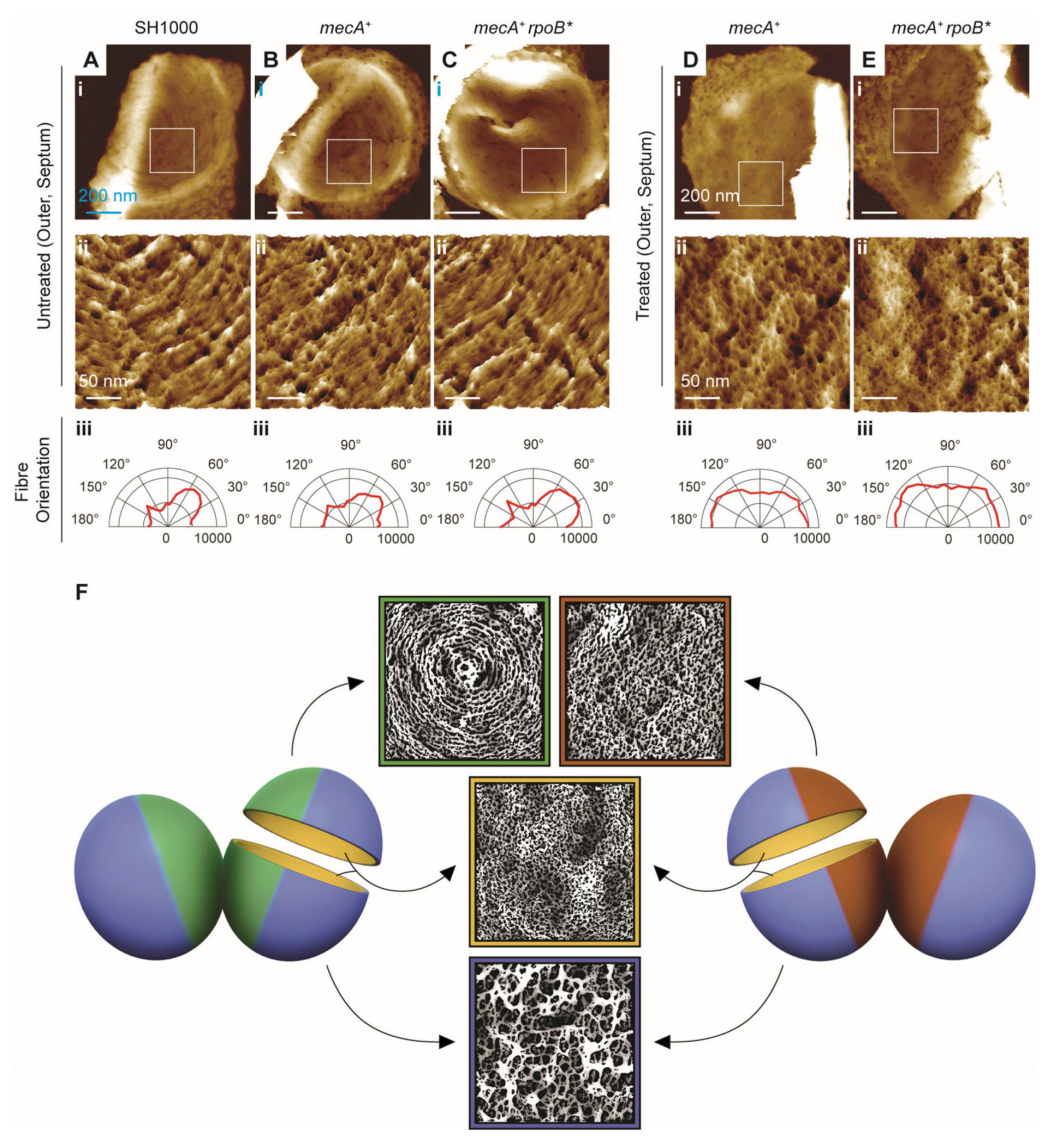
Methicillin treatment of MRSA alters the architecture of the cell wall. From left to right, (**A-C**) show the outer surfaces of newly revealed septa, in samples of isolated sacculi of untreated (**A**) SH1000, (**B**) *mecA*^+^, and (**C**) *mecA*^+^ rpoB* respectively. (**D-E**) Show the outer surface of the newly revealed septa of (**D**) *mecA*^+^ and (**E**) *mecA*^*+*^ rpoB* treated with methicillin (1.5 and 25 μg ml^-1^ respectively). In all columns: (i) shows an individual fragment of sacculus corresponding to the outer surface of the septum. Topographical height (z) range presented in each of these images (from left to right) is 140, 140, 150, 120, and 185 nm. (ii) Shows pseudo-three dimensional (3D) high resolution AFM images of the sections indicated by the white boxes in (i). Topographical height (z) range presented in each of these images (from left to right) is 7.5, 10, 7.5, 12, and 20 nm. (iii) Represents the combined angular histogram of fibre orientation of AFM high-resolution images similar to those in (ii). The fibre orientation analysis method used for the orientation detection is described in the Materials and Methods section. **(F)** Shows an interpretative diagram of different architectures (concentric rings, dense mesh, and open mesh) observed by high-resolution AFM on different surfaces (outer surface of newly revealed septa, inner surface of the septa, and outer surface of cell periphery) of untreated (left-hand side) and antibiotic treated (right-hand side) MRSA cell wall. The green colour represents the concentric rings associated with the outer surface of the septum of untreated cells, blue colour shows the open mesh at the cell periphery, yellow colour depicts the dense mesh on the inner wall of the cell and lastly the brown colour represents the dense mesh on the outer surface of the septum after treatment with methicillin. The modified AFM images in Fig. 1F span 400 nm by 400 nm in x and y dimension.

AFM was used to analyse the nanoscale, PG architecture, where in all cases at least 20 individual sacculi (i.e. purified cell wall fragments) were examined (see Materials and Methods). AFM analysis (Fig. 1A-B; and fig. S1A-B and S2A-B) showed that low-level resistant MRSA (SH1000 *mecA*^+^ (hereafter designated *mecA*^+^); MIC 2 μg ml^-1^), in the absence of methicillin, resembled its sensitive parent (SH1000; MIC 0.25 μg ml^-1^). In both cases, the inner surface of the cell wall in all areas consisted of a dense mesh of PG, the outer surface of the septum, newly exposed after division, exhibited the characteristic septal PG concentric-ring architecture, and the PG at the outer surface of the cell, away from the most recent site of division, consisted of an open mesh structure (6) (Fig. 1A-B; and fig. S1A-B, S2A-B). We quantified the orientation of individual glycan strands for strains SH1000 and *mecA*^*+*^ in the absence of antibiotic using a custom-made automated image analysis. This revealed that in both cases the outer surface of the septum exhibited a prominent peak in the circumferential direction that is consistent with the concentric-ring architecture (Fig. 1Aiii, Biii). However, no PG concentric rings were apparent at the outer surface of the septum of *mecA*^*+*^ in the presence of 1.5 μg ml^-1^ methicillin (sub-MIC for *mecA*^*+*^). Rather, the outer surface of the septum appeared as a dense mesh structure (Fig. 1Di-iii), while the inner surface displayed a large proportion of long glycan strands that were oriented near the septal centre (fig. S1Dii, see the long orange-brown coloured-fibres in fig. S1Diii). Furthermore, the cell wall was thinner after treatment with methicillin (fig. S1F). Under the same conditions (1.5 μg ml^-1^ methicillin), the parental stain, SH1000, died and cell wall spanning holes were apparent (15) (fig. S2F-H). The cell wall architecture of the high-level MRSA strain (SH1000 *mecA*^*+*^ rpoB* (hereafter designated *mecA*^*+*^ rpoB*); MIC ≥256 μg ml^-1^), which possessed both *mecA* and the *pot* mutation, rpoB* coding for a variant of the RNA polymerase β subunit RpoB(H929Q) (14), resembled that of the parental strain (*mecA*^+^) in the absence of antibiotics (Fig. 1C; and fig. S1C and fig. S2C). When treated with 25 μg ml^-1^ methicillin (sub-MIC for this strain but sufficient to kill both SH1000 and *mecA*^*+*^) the inner surface of the cell wall maintained a dense network of PG mesh, without the appearance of perforating holes (fig. S1Eii). However, in the large majority of cases, the septa were thickened with a distinct protuberance, or lump, at the centre (fig. S1Ei). Importantly, although *mecA*^*+*^ rpoB* was able to grow and divide in the presence of methicillin, there was a total absence of the PG concentric-ring structure on the outer surface of newly divided cells (Fig. 1E). Septal PG concentric rings are a defining feature of PG architecture in several gram-positive bacteria (6, 16). Instead of PG concentric rings, the outer surface of septa obtained from methicillin-treated *mecA*^*+*^ rpoB* consisted of a disordered, dense mesh with small pore size (Fig. 1E). As in the absence of antibiotics, the outer surface of the rest of the cell periphery appeared as a more open mesh with larger pore size (fig. S2E and S2I). This open mesh structure is derived from the dense mesh rather than from the concentric ring structure, which remodels as cells divide in different planes during subsequent division rounds (16). An interpretative diagram illustrating these observations is shown in Fig. 1F.

We then used the clinical, high-level, MRSA strain COL (SCC*mec* Type I), which possesses both the *mecA* gene and produces a variant RpoB(A798V, S875L) (14) (MIC ≥256 μg ml^-1^) to determine whether the resistance-associated PG architectural changes described above (absence of septal PG concentric rings, retention of PG dense mesh without perforating holes) are a common feature of MRSA cells under antibiotic stress. The COL cells were smaller than SH1000 (average cell volume 0.69 ± 0.14 vs 1.22 ± 0.31 μm^3^) as were the cells of *mecA*^*+*^ rpoB* (average cell volume 0.60 ± 0.20 μm^3^, fig. S4D). Without antibiotics, COL displayed septal PG concentric rings (fig. S3A), whereas in the presence of 25 μg ml^-1^ methicillin (sub-MIC), the septal PG of COL exhibited no concentric rings, but rather a disordered, dense mesh, at the septal outer surface (fig. S3G). Treatment of *mecA*^*+*^, *mecA*^*+*^ rpoB* and COL with sub-MIC concentrations of antibiotics (1.^5^, 25, and 25 μg ml^-1^, respectively) led to high levels of PG synthesis at the septum (as observed by ADA-DA incorporation), an increase in cell volume and septal abnormalities observed by fluorescence microscopy and transmission electron microscopy (TEM) (fig. S4).

To demonstrate the wider applicability of our findings we then analyzed representatives of different MRSA lineages and SCC*mec* types (*SCCmec* II (Mu50, MRSA252), III (TW20) and IV (USA300, EMRSA15)) *(3, 17-20)*. All strains had methicillin MICs of >256 μg ml^−1^ apart from EMRSA15 and USA300 (MIC 64 and 1-2 μg ml^−1^, respectively) (Table S1). High-level MRSA derivatives (MIC >256 μg ml^−1^), of the latter two strains, designated USA300 (HL) and EMRSA15 (HL), were selected by directed evolution on oxacillin gradient plates (see Materials and Methods).

AFM analysis of the clinical strains and high-level resistant derivatives was carried out in the absence and presence of 25 μg ml^−1^ methicillin (sub-MIC; fig. S3). All untreated strains had septal PG concentric rings at the outer face of the septum (fig. S3). In the presence of 25 μg ml^-1^ methicillin (sub-MIC), the septal PG of COL, EMRSA15 (HL) and USA300 (HL) had a disordered, dense mesh, at the septal outer surface but Mu50, MRSA252 and TW20 had occasional (10 - 30% of septa) residual PG orientation. Growth of Mu50, MRSA252, and TW20 in 50 μg ml^-1^ methicillin (sub-MIC) gave rise to disordered mesh at the septal outer surface (fig. S3). Thus, similar adaptations in septal PG architecture in response to antibiotic challenge are conserved across MRSA strains (Fig. 1F).

Thus, even though PBP2a, in MRSA backgrounds permits growth and division in the presence of antibiotics, it leads to profound changes to cell wall architecture. This raises the questions as to how PBP2a complements the loss of both essential PBP1 and PBP2 transpeptidase activities, and also how high-level MRSA is able to divide?

### Mode of cell division underpins high-level MRSA

**Fig. 2:**
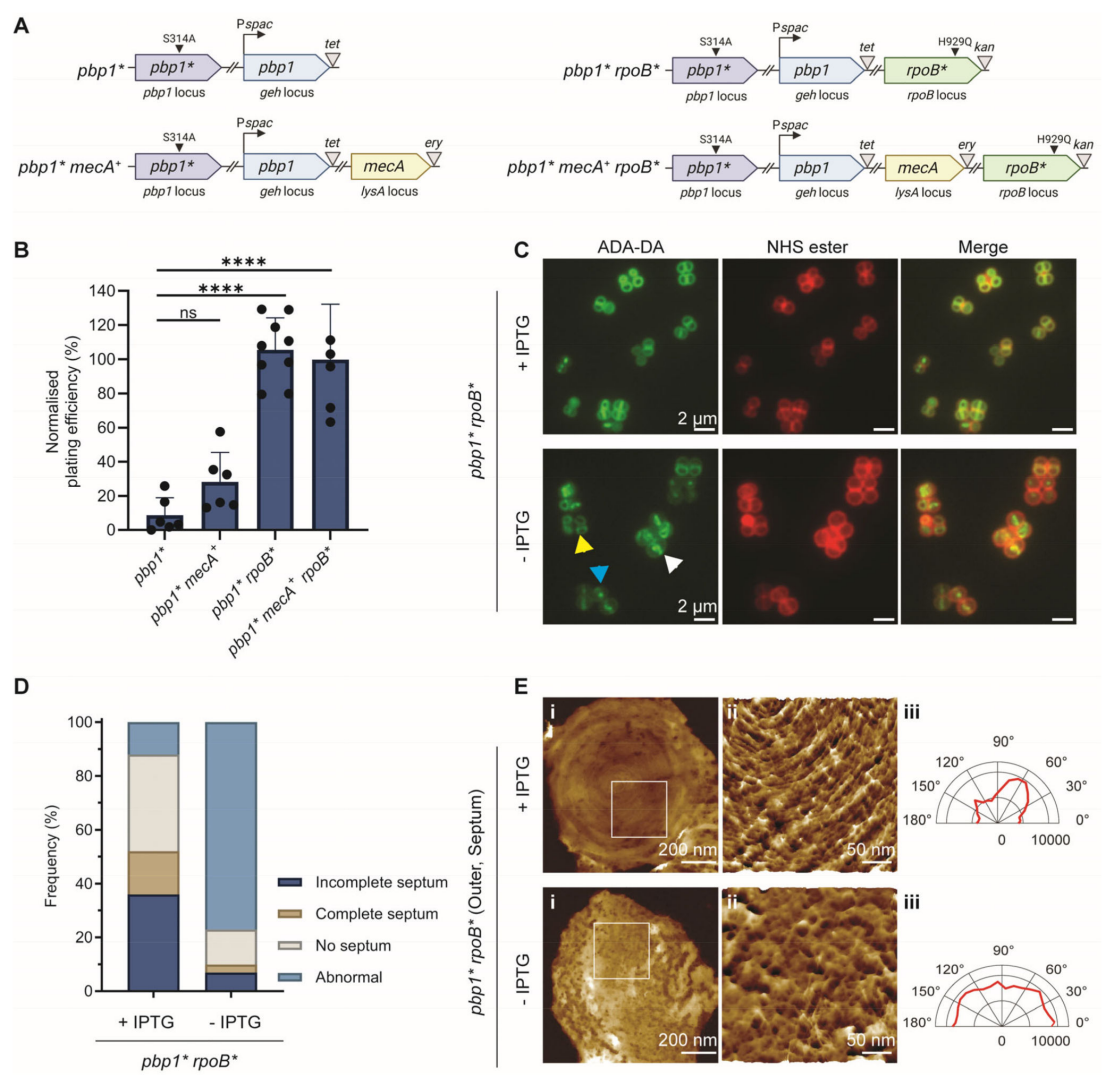
Loss of PBP1 transpeptidase activity can be compensated for by rpoB* but not *mecA*. (**A**) Representation of pbp1* genetic constructs. An ectopic *pbp1* copy, at the lipase (*geh*) locus is controlled by the P*spac* promoter. The *pbp1* gene at its native locus has a point mutation (940T>G) resulting in inactivation of transpeptidase activity (S314A, pbp1*). The *mecA*^+^ gene is expressed from its native promoter at the *lysA* locus. In rpoB*, a point mutation results in an amino acid substitution (H929Q) in RpoB. *tet, ery* and *kan* represent tetracycline, erythromycin and kanamycin resistance cassettes, respectively. The graphics were created with BioRender.com. (**B**) Plating efficiency of pbp1*, pbp1* mecA^+^, pbp1* rpoB* and pbp1* mecA^+^ rpoB* without IPTG. Plating efficiencies were compared to controls grown with IPTG, using a one-way ANOVA with Dunnett’s multiple comparison test (ns, not significant; ****, *P <* 0.0001). Error bars show mean ± standard deviation (SD). (**C**) Fluorescence microscopy images of pbp1* rpoB* grown +/-IPTG for 4 h, labelled with ADA-DA (5 min) and then NHS-ester Alexa Fluor 555 to image nascent PG and cell wall, respectively. Images are z stack average intensity projections. Scale bars = 2 μm. (**D**) Quantification of cellular phenotypes based on ADA-DA incorporation in pbp1* rpoB* incubated with IPTG (+) or without IPTG (-), n = 511 and 654 (respectively). Examples of cells classified as abnormal with misshapen septal rings (yellow arrowhead), accumulation of ADA-DA at septal centre, ‘plug’ (blue arrowhead) and mislocalized ADA-DA incorporation (white arrowhead) are shown C. **(E**) AFM images of newly exposed outer surface of the septum after cell division of pbp1* rpoB* grown +/-IPTG for 4 h, reveal lack of concentric-ring structures in -IPTG. (i) Representative outer septal surfaces with height (z) range of 120 nm and the HS applies to both. (ii) Shows pseudo-3D AFM high resolution images of the region within the white box in (i). Topographical height (z) range (top) = 9.5 nm, and HS (bottom) = 21 nm. (iii) Represents the combined angular histogram of fibre orientation of AFM high resolution images similar to those in (ii).

We have recently suggested that the *S. aureus* septal PG concentric rings are due to PBP1 transpeptidase activity (8). Methicillin sensitive *S. aureus* (MSSA) specifically lacking PBP1 transpeptidase activity is not viable and exhibits aberrant septa (8). However, a high-level MRSA strain with the same site-directed inactivation of PBP1 transpeptidase activity can grow (8), suggesting that PBP2a complements the lack of PBP1 activity, but perhaps without the ability to construct the septal PG concentric-ring structures. We therefore constructed a set of otherwise isogenic strains where, in the absence of the inducer IPTG, only PBP1 without transpeptidase activity (PBP1*) was expressed (Fig. 2A; and fig. S5A and B). Wholly unexpectedly, the presence of PBP2a in this background SH1000 Pspac-pbp1 pbp1* mecA^*+*^ (hereafter designated pbp1* mecA^*+*^) did not complement the loss of PBP1 transpeptidase activity, demonstrating that PBP2a cannot substitute for the essential transpeptidase function of PBP1 (Fig. 2B). Conversely, a single point mutation in *rpoB* (resulting in amino acid replacement H929Q; rpoB*), that is required for MRSA with high-level resistance (14), was able to entirely restore the ability of PBP1* to grow in the absence of PBP2a (Fig. 2B; and fig. S5C and D). Growth of Pspac-pbp1 pbp1* rpoB* (hereafter designated pbp1* rpoB*) without IPTG was associated with septal abnormalities, an increase in cell volume, and alterations to PG synthesis (Fig. 2C and D; and fig. S5E-F and S6A), similar to high-level MRSA grown in the presence of antibiotics (fig. S4B).

AFM analysis of the PG architecture of pbp1* rpoB* with IPTG (PBP1 transpeptidase activity present) revealed open mesh on outer surfaces and septal PG concentric rings as expected for a wild type strain (Fig. 2E (+IPTG); Fig. S6B to D). However, growth without IPTG (no PBP1 transpeptidase activity) led to the concentric rings at the septal surface being replaced by a disordered, dense mesh with random glycan strand orientation (Fig. 2E (-IPTG); and fig. S6E to G). Although rpoB* complemented the absence of PBP1 transpeptidase activity, neither rpoB* nor PBP2a, or both combined, could rescue cells lacking the PBP1 protein (fig. S7), consistent with the physical presence of PBP1 being necessary for cell division complex assembly. Therefore, the septal PG ring architecture associated with conventional cell division requires the essential transpeptidase activity of PBP1, but *S. aureus* can adopt an alternative division mode facilitated by rpoB* when PBP1 transpeptidase activity is lost (either by mutation or antibiotic addition; Fig. 1F). This fundamentally different mode of cell division, which lacks the canonical septal PG concentric-ring architecture, is exploited in high-level MRSA, where rpoB* in combination with *mecA* allows division in the presence of antibiotics.

### Dual mechanisms for high-level MRSA

**Fig. 3:**
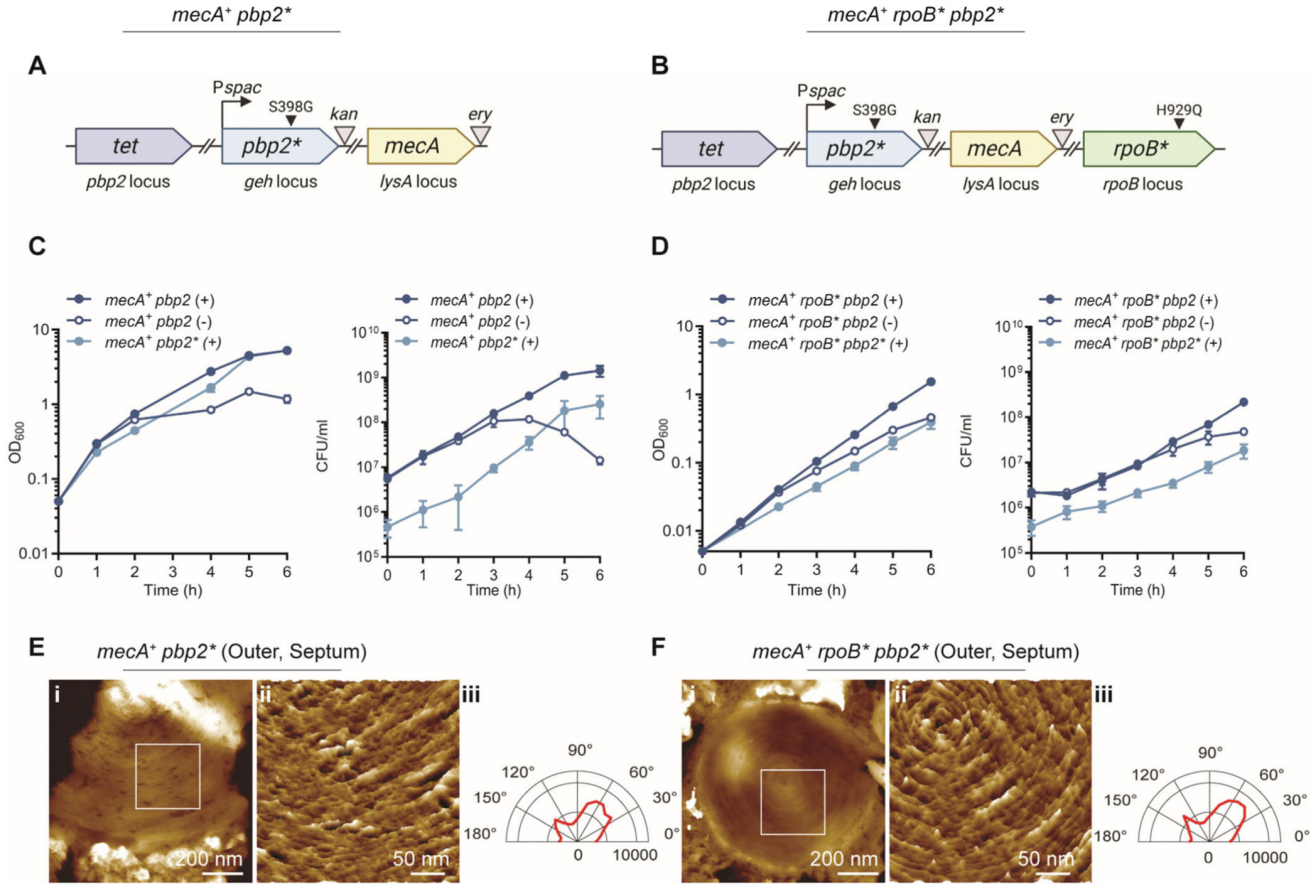
Loss of PBP2 transpeptidase activity can be compensated for by *mecA* but not rpoB*. (**A-B**) Schematic representation of *mecA*^+^ pbp2* (SJF5807) and *mecA*^*+*^ rpoB* pbp2* (SJF5809) genetic constructs. A copy of *pbp2* with an inactive transpeptidase domain (pbp2*, 1191-1192TC>GG, S398G) was placed under the control of the P*spac* promoter at the lipase (*geh*) locus of SH1000, *pbp2* at its native locus was then deleted (marked with *tet)*. In both strains, a copy of a *mecA* gene expressed from its native promoter was located at the *lysA* locus. In *mecA*^+^ rpoB* pbp2* (SJF5809) the *rpoB* gene has a point mutation which results in H929Q (rpoB*). *ery* and *kan* represent erythromycin and kanamycin resistance cassettes, respectively. The graphics in (**A-B**) were created with BioRender.com. (**C**) Growth curves of *mecA*^+^ *pbp2* (SJF5663) grown in the presence (+) or absence (-) of IPTG, and *mecA*^+^ pbp2* (SJF5807) (+ IPTG). (**D**) Growth curves of *mecA*^+^ rpoB* pbp2 (SJF5674) grown in the presence (+) or absence (-) of IPTG, and *mecA*^+^ rpoB* pbp2* (SJF5809) (+ IPTG). (**E-F**) AFM images of the newly revealed outer surface of septa after cell division of *mecA*^+^ pbp2* (SJF5807) and *mecA*^*+*^ rpoB* pbp2* (SJF5809), respectively. In both **E** and **F**, (i) shows the outer surface of a representative septum. Topographical height (z) range of 130 nm applies to both. (ii) Shows a pseudo-3D high resolution image of the region within the white box in (i). Height range are 12 nm for **E**(ii) and 7 nm for **F**(ii). (iii) Represents the combined angular histogram of fibre orientation of AFM high resolution images similar to images in (ii).

High-level MRSA requires two factors; the presence of PBP2a and a potentiator (*pot*) mutation (as provided by rpoB*) (13, 14). For high-level MRSA to grow and divide in the presence of β-lactam antibiotics, the essentiality of PBP1 and PBP2 transpeptidase activities must be circumvented or enzymatically complemented. Previous studies report that in strain COL the transpeptidase activity of PBP2 can be complemented by the presence of PBP2a (21, 22). However, growth of a COL derivative lacking PBP2 protein is impaired and does not exhibit antibiotic resistance (22). This is because PBP2 transglycosylase activity is required to act cooperatively with PBP2a (22). COL also harbours potentiator rpoB* mutations (A798V, S875L) required for high-level resistance (14). To determine whether there are two co-dependent mechanisms that in combination lead to high-level MRSA we investigated the effect of *pbp2* mutations. As expected from previous reports (21, 22) PBP2 is essential and PBP2a and/or rpoB* (H929Q) could not compensate for the loss of PBP2 protein in terms of plating efficiency and growth (fig. S8A-C). When PBP2 was depleted, with or without the presence of PBP2a, *S. aureus* stopped dividing, exhibiting decreased septal PG incorporation and altered septal morphology (fig. S9). Loss of PBP2 also led to a decrease in cell size (fig. S9H). Depletion of PBP2 in rpoB* or *mecA*^*+*^ rpoB* led to lower growth, decreased septal PG incorporation, altered septal morphology, and death (fig. S9C, D, and G). We could not create PBP2* (transpeptidase mutant) strains in either the parental SH1000 or rpoB* backgrounds, indicating its essentiality. However, strains where only PBP2* is present were viable in both *mecA*^*+*^ and *mecA*^*+*^ rpoB* (Fig. 3A to D). Both the PBP2 and PBP2* constructs were verified by Western blot and Bocillin labelling (fig. S8D and E). Both strains with PBP2* were able to grow with near parental (*mecA*^*+*^ and *mecA*^*+*^ rpoB*, respectively) cell morphology (fig. S9E and F). All PBP2 and PBP2* constructs demonstrate a diminished cell size compared to SH1000 (fig. S9H). Expression of PBP2* (lacking PBP2 transpeptidase activity) in the *mecA*^*+*^ or *mecA*^*+*^ rpoB* backgrounds resulted in septa that exhibited the typical PG concentric-ring architecture, with strands preferentially oriented in the circumferential direction (Fig. 3E and F and fig. S10). We conclude that neither PBP2 nor PBP2a are responsible, even in part, for the PG septal concentric rings associated with conventional cell division. Therefore, there are two factors required for high-level MRSA: (i) PBP2a replaces the essential transpeptidase activity of PBP2, and (ii) a *pot* mutation (e.g. rpoB*) permits cell division without PBP1 transpeptidase activity.

### Potentiator mutations converge on nucleotide signalling

Mutations in *rpoB* and *rpoC* have been associated with clinically important high-level MRSA strains and the conversion from hetero-to homogeneous resistance (13, 14, 23, 24). Other *pot* mutations, such as *rel, clpXP, gdpP, pde2* and *lytH* have been uncovered in laboratory studies and in some cases clinically (13). Whilst other mutations enhanced the MIC of *mecA*^*+*^, tested in our defined SH1000 background with a single copy of *mecA* in the chromosome, only *rpoB* and *rel* led to high-level resistance (table S1; MIC ≥256 μg ml^-1^).

**Fig. 4:**
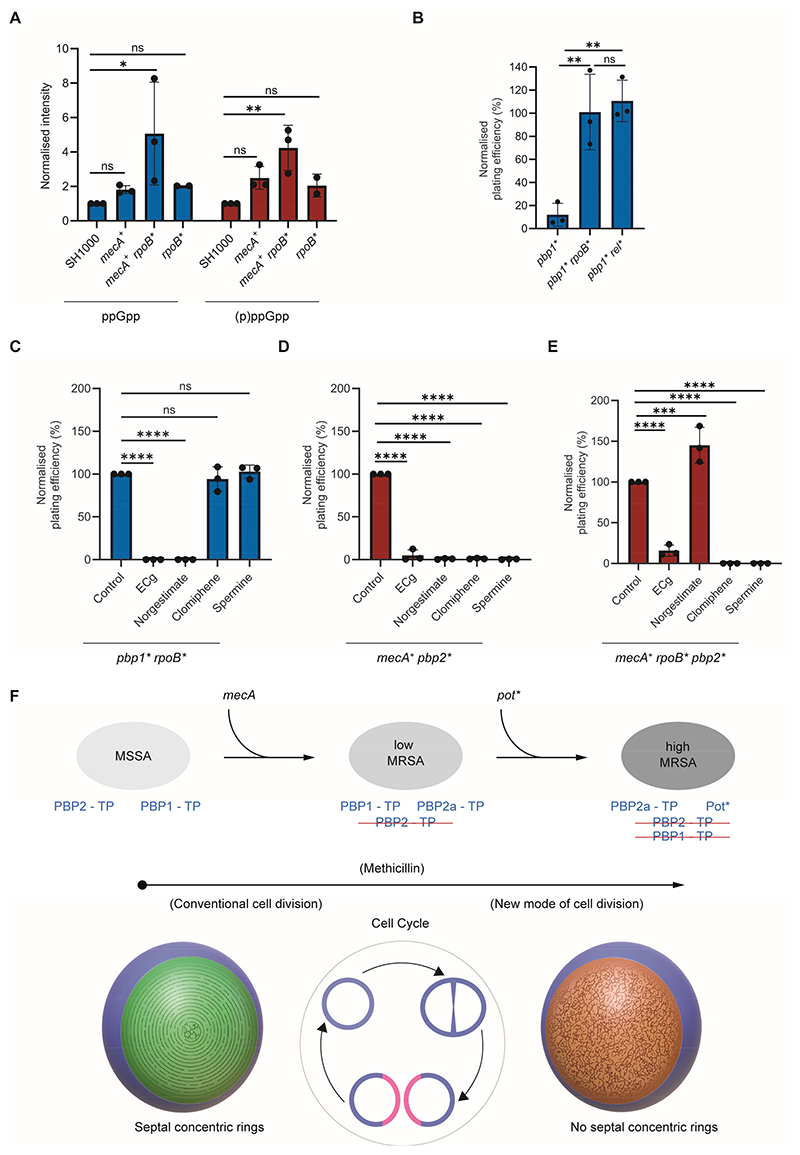
Dual pathways to high-level MRSA. (**A**) Measurement of ppGpp and (p)ppGpp levels in SH1000, *mecA*^+^, rpoB* and *mecA*^+^ *rpoB*, normalised to SH1000 and compared using one-way ANOVA with Dunnet’s multiple comparison test (ns, not significant; *, *P <*0.05; **, *P <*0.01). *P* values from left to right: 0.8727, 0.0425, 0.8290, 0.1470, 0.0051 and 0.4317. Error bars show the mean ± SD. (**B**) Plating efficiency of pbp1*, pbp1* rpoB* and pbp1* rel without IPTG. Plating efficiency values were compared to controls with IPTG, using one-way ANOVA with Tukey’s multiple comparison test (**, left to right *P =* 0.0049 and 0.003). Error bars show mean ± SD. (**C-E**) Plating efficiency of (**C**) pbp1* rpoB*, (**D**) *mecA*^+^ pbp2*and (**E**) *mecA*^+^ rpoB* pbp2* without IPTG supplemented with ECg, norgestimate, clomiphene or spermine. Data were compared to no inhibitor plates (Control) using a one-way ANOVA with Dunnett’s multiple comparison test (ns, not significant; ****, *P <*0.0001; ***, *P =* 0.0004). Error bars show mean ±SD from three independent biological repeats. (**F**) Model for high-level MRSA development via acquisition of *mecA* and *pot* mutations (including rpo* and rel*), resulting in low-level (low) and subsequently high-level (high) resistance. In MSSA, without methicillin, PBP1 and PBP2 transpeptidases are active. In low-level MRSA, at intermediate methicillin levels sufficient to kill MSSA, PBP2 transpeptidase is inhibited but complemented by PBP2a. In high-level MRSA, at methicillin levels sufficient to kill MSSA and low-level MRSA, PBP2 and PBP1 transpeptidases are inhibited but complemented by PBP2a transpeptidase and Pot*, respectively. PBP1 transpeptidase is responsible for the characteristic septal PG concentric rings, during conventional cell division (green regions on blue cell background). In high-level MRSA, in the presence of methicillin, septal PG concentric rings are replaced by mesh (brown regions on blue cell background), revealing a novel mode of cell division requiring both PBP2a and Pot*.

The *rel* gene encodes a key component of the stringent response (25) and whilst the gene is conditionally essential, the *pot* mutant strain (rel*) has a C-terminal truncation in the regulatory domain of the Rel protein, and likely increases (p)ppGpp levels (26). The stringent response has been previously implicated as having a major role in potentiating high-level MRSA (27) and here we found the presence of *mecA*^*+*^ rpoB* led to a significant increase in the levels of the stringent response signalling molecules ppGpp and pppGpp (Fig. 4A). To determine the relationship between the stringent response and the dual pathways to high-level MRSA we investigated its ability to compensate for the loss of PBP1 transpeptidase activity (Fig. 4B). The rel* mutation was as effective as rpoB* in compensating for the absence of PBP1 transpeptidase activity as judged by measurement of plating efficiency (Fig. 4B), implicating the stringent response in the ability to grow and divide without septal PG concentric rings.

### Therapeutic development for MRSA

To counter the emergence of MRSA, compounds have been identified that resensitize these strains to β-lactams (28). These include clomiphene (29) and norgestimate (30), as well as natural products including epicatechin gallate (ECg) (31) and spermine (32). Their mode of action is mostly unknown and so we tested their effect, at concentrations that resensitize *mecA*^*+*^ rpoB* and the other clinical MRSA strains to oxacillin but do not inhibit growth without antibiotic (see Materials and Methods) (Fig. 4C to E). Clomiphene and spermine did not inhibit the plating efficiency of pbp1* rpoB* but did for both *mecA*^*+*^ pbp2* and *mecA*^*+*^ pbp2* rpoB*, suggesting a link to the activity of PBP2a. Norgestimate impaired the plating efficiency of pbp1* rpoB* and *mecA*^*+*^ pbp2* but not *mecA*^*+*^ pbp2* rpoB*, demonstrating a potential cross-talk between the co-dependent pathways (i.e., acquisition of *mecA* and a *pot* mutation) that lead to resistance. ECg inhibited the plating efficiency of all three strains indicating that it may affect an Aux factor required under all conditions. These observations further differentiate the two resistance pathways and provide specific interventions able to dissect the new mode of cell division uncovered here.

## Discussion

We have revealed that the high-level resistance to β-lactam antibiotics exhibited by some MRSA strains is linked to an alternative mode of cell division set within the context of wider physiological adaptations (i.e., increased ppGpp and pppGpp) (Fig. 4F). The development of high-level MRSA is a two-step process in which PBP2a compensates for the lack of native PBP2 transpeptidase activity in the presence of β-lactam antibiotics (22). PBP2 is an essential enzyme that is required for the synthesis of the dense mesh PG on the inside of the cell wall at both the septum and the cell periphery. It is therefore the major PBP in terms of bulk PG synthesis. PBP2a cannot compensate for the lack of PBP2 protein (specifically its transglycosylase activity (22)). However, as PBPs can form dimers (33), PBP2/2a heterodimers could allow both the multiple protein interactions of PBP2 (34) and PBP2a transpeptidase activity required for PG synthesis. PBP1 has essential transpeptidase activity and operates with its cognate transglycosylase FtsW (35). Here we show that PBP1 activity is responsible for the formation of the concentric rings that are characteristic of septal PG. PBP2a cannot compensate for the lack of PBP1 activity but *pot* mutations can. The *pot* mutations permit successful cell division without septal PG rings in the presence of high levels of antibiotics (Fig. 4F). This compensatory mechanism does not involve a replacement of PBP1 activity but rather physiological adaptations that allow division without it. A question arises as to whether the ability to divide without septal PG concentric rings in high-level MRSA strains evolved specifically, in the context of antibiotic use, or whether it is part of a wider physiological capability that is deployed under stressful conditions? Mutations in *rpo* genes are often found associated with antibiotic and stress resistance in *S. aureus* and many other organisms (24, 36, 37). A survey of 1,429 MRSA (ST22) clinical strains revealed that ∼10% had at least one point mutation in genes coding for core RNAP subunits or σ factors (24). The current study now links these mutations to the widely conserved stringent response, which is a key component in bacterial responses to stress and growth perturbations (25). Our rpoB* strains exhibit lower growth rates compared to parental strains (14), which could, at least in-part, facilitate the alternative mode of division.

Given the array of MRSA SCC*mec* types and clonal lineages, it is likely that the effects of *pot* factors, such as rpo*, are influenced by the genetic background (13). This provides both complexity in unravelling the interplay between *pot* and *aux* factors but also an opportunity to establish those common, underlying principles that underpin resistance. The resensitizing agents also provide avenues to probe underlying molecular mechanisms. Our study has revealed insights into antibiotic resistance and facets of cell division in *S. aureus*. It is by studying these processes in tandem that we can understand basic mechanisms of the bacterial cell cycle and reveal ways to control antibiotic resistance.

## Supplementary Material

Supplementary Materials

## Data Availability

The data that support the findings of this study are available in the Online Research Data (ORDA) figshare from the University of Sheffield with the identifier (38).

## One Sentence Summary

High-level resistance in MRSA requires two pathways that reveal a novel cell division mode.
