# Supplementary material for: Two co-dependent routes lead to high-level MRSA
Source: Science. Author manuscript; Available in PMC 2025 Jul 5. (PMC7617827; doi:10.1126/science.adn1369)
Supplement: Supplementary Materials [file EMS206345-supplement-Supplementary_Materials.pdf]

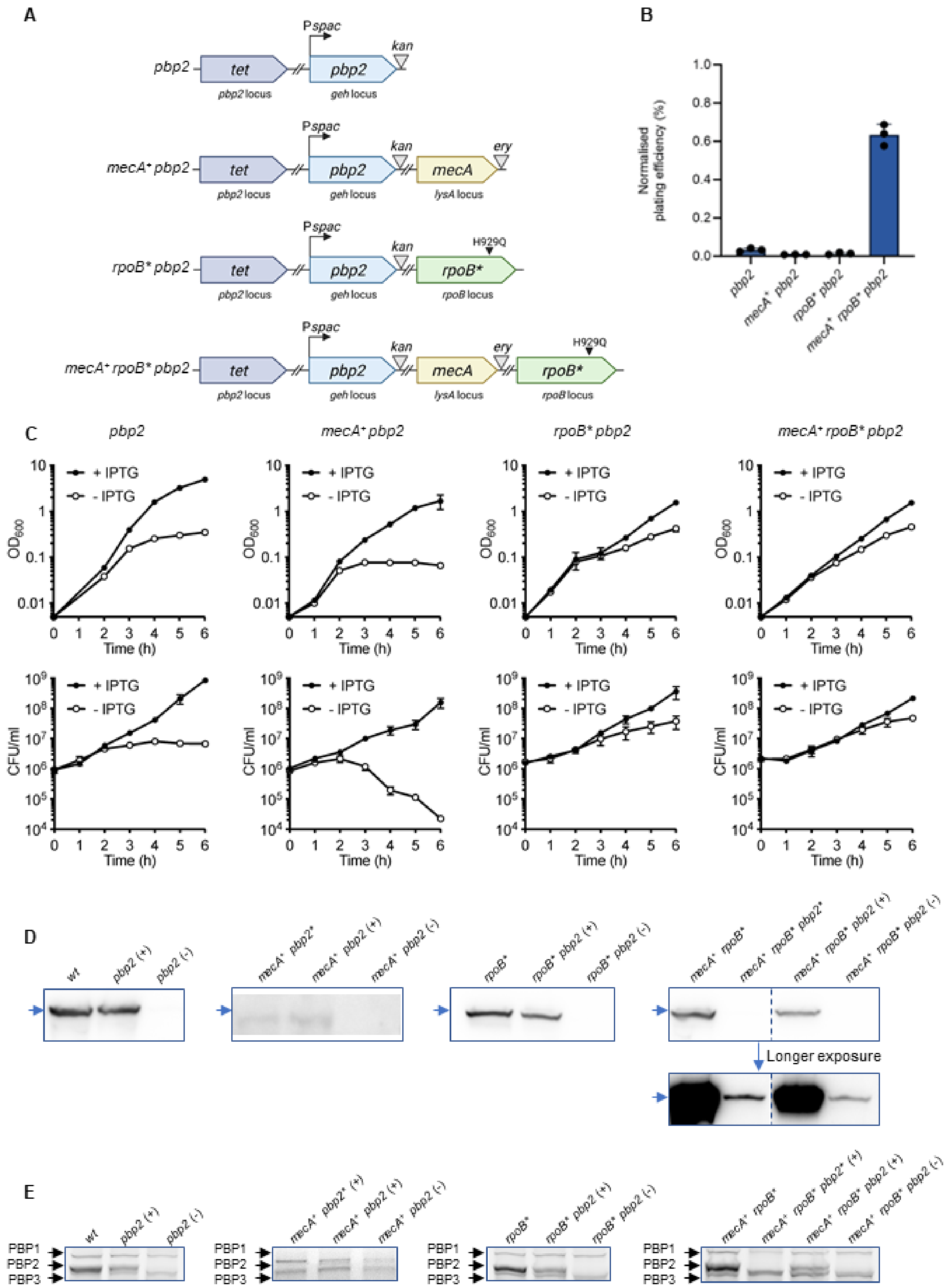

Fig. S8.

Analysis of the role of PBP2 in growth. (**A**) Schematic representation of the *pbp2* genetic constructs. An ectopic copy of *pbp2* was placed under the control of the Pspac promoter at the SH1000 lipase (*geh*) locus, while the gene in the native *pbp2* locus was deleted (marked with *tet*). In *mecA*$^+$ *pbp2* and *mecA*$^+$ *rpoB** *pbp2* a copy of a *mecA* gene expressed from its native

promoter was located at the *lysA* locus. In *rpoB** *pbp2* and *mecA*$^+$ *rpoB** *pbp2* the *rpoB* gene has a point mutation which results in a single amino acid change (H929Q) in the RNA polymerase β subunit (*rpoB**). The graphics were created with BioRender.com. (**B**) Plating efficiency of the derivatives *pbp2*, *mecA*$^+$ *pbp2*, *rpoB** *pbp2* and *mecA*$^+$ *rpoB** *pbp2* grown in the absence of IPTG. Plating efficiency values were compared with the control groups grown in the presence of IPTG. Data represent the mean ± SD. (**C**) Growth curves of SH1000 derivatives *pbp2*, *mecA*$^+$ *pbp2*, *rpoB** *pbp2*, and *mecA*$^+$ *rpoB** *pbp2* grown in the presence or absence of IPTG (+ IPTG and - IPTG, respectively). Data represent the mean ± SD. Error bars that are smaller than the data point symbols are not shown. (**D**) Immunoblots, analysed using anti-PBP2 antibody, of whole cell lysates of SH1000 (wt) and *mecA*$^+$ *rpoB**, *rpoB**, *pbp2*, *mecA*$^+$ *pbp2*, *rpoB** *pbp2* and *mecA*$^+$ *rpoB** *pbp2* derivatives grown in the presence (+) or absence (-) of IPTG for 4 h. *mecA*$^+$ *pbp2** and *mecA*$^+$ *rpoB** *pbp2** were grown in the presence of IPTG throughout. Expected PBP2 and PBP2* sizes = 80 kDa are indicated (blue arrowhead). (**E**) BocillinFL gel-based analysis of PBPs in SH1000 and *mecA*$^+$ *rpoB**, *rpoB**, *pbp2*, *mecA*$^+$ *pbp2*, *rpoB** *pbp2,* and *mecA*$^+$ *rpoB** *pbp2* grown in the presence (+) or absence (-) of IPTG for 4 h. *mecA*$^+$ *pbp2** and *mecA*$^+$ *rpoB** *pbp2** were grown in the presence of IPTG throughout. Data are representative of two (**D** and **E**) and three (**B** and **C**) independent biological experiments.

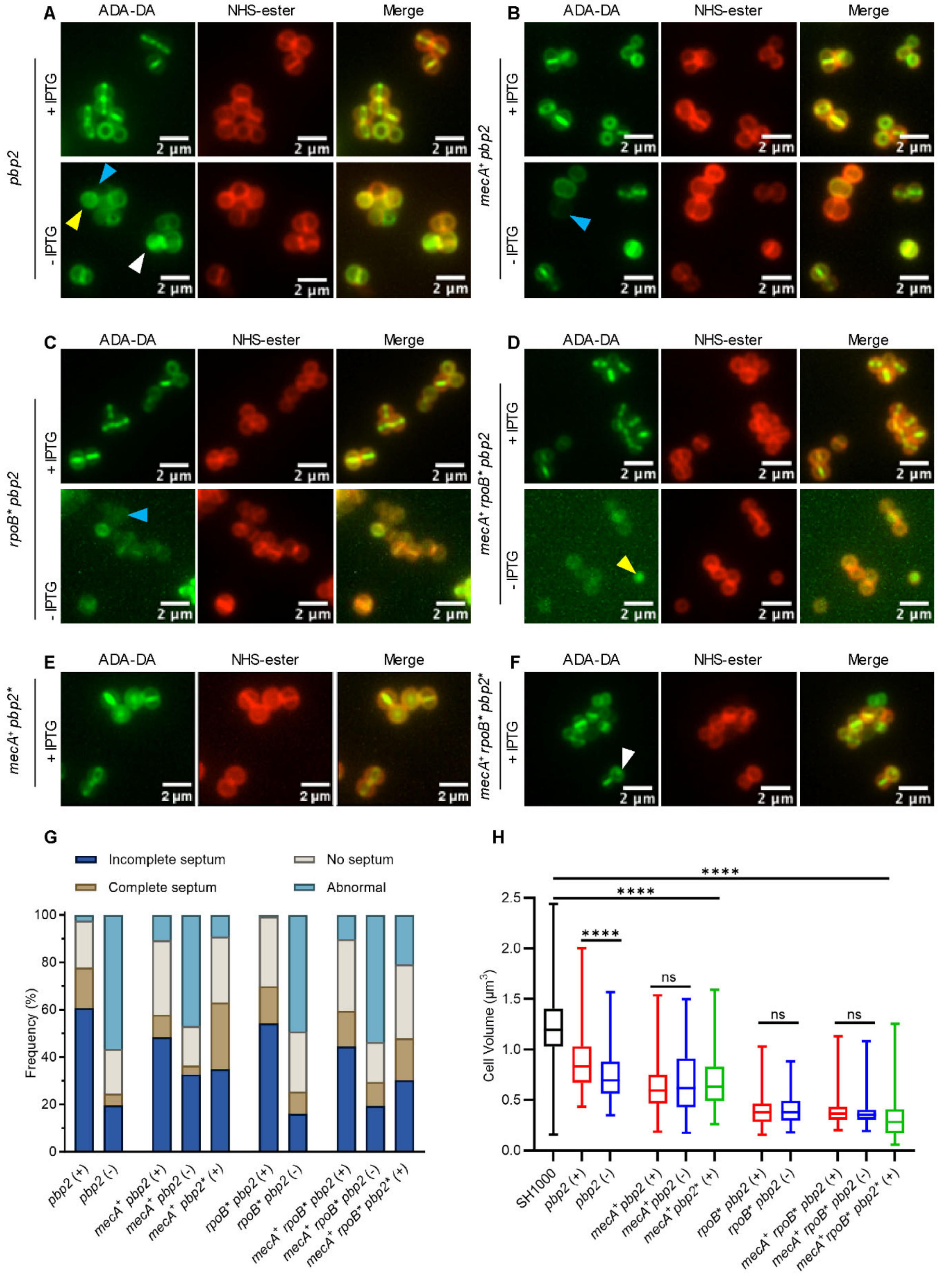

Fig. S9.

Role of PBP2 in cell morphology. (**A-F**) Fluorescence microscopy images of the SH1000 derivatives *pbp2* (**A**), *mecA*$^+$ *pbp2* (**B**), *rpoB** *pbp2* (**C**) and *mecA*$^+$ *rpoB** *pbp2* (**D**) grown in

the presence (+ IPTG) or absence (- IPTG) of the inducer for 4 h. *mecA*$^{+}$ *pbp2** (**E**) and *mecA*$^{+}$ *rpoB* pbp2** (**F**) were grown in the presence of IPTG at all times. All strains were incubated for 5 min with ADA-DA clicked to Atto488 to show nascent PG, and counter labelled with NHS-ester Alexa Fluor 555 to image the cell wall. Images are average intensity projections of z stacks. Cells with dispersed, apparent ADA-DA incorporation (yellow arrowheads), no ADA-DA incorporation (blue arrowheads) or mislocalized ADA-DA incorporation (white arrowheads) are examples of cells that were classified as abnormal in panel **G**. (**G**) Quantification of cellular phenotypes based on ADA-DA incorporation in **A-F**. From left to right, n = 247, 235, 261, 288, 237, 273, 299, 309, 278 and 305. (**H**) Cell volumes of strains in **A-F** measured by fluorescence microscopy after NHS-ester Alexa Fluor 555 labelling. Number of cells analysed for each sample was $n \geq 300$. Data are representative of three independent biological experiments.

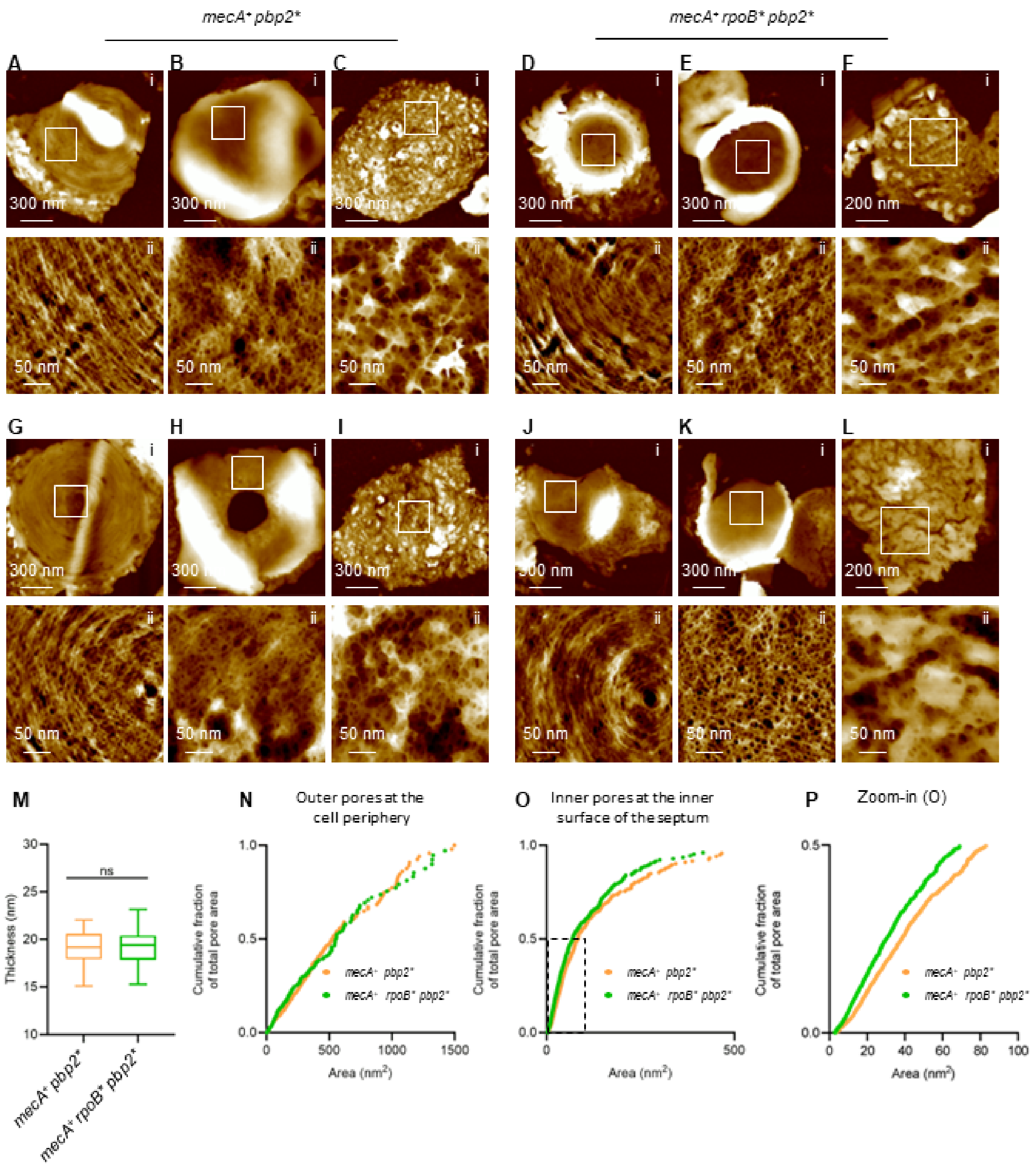

Fig. S10.

Surface-dependent nanoscale architecture of *mecA*$^+$ *pbp2** and *mecA*$^+$ *rpoB* pbp2** revealed by AFM. AFM topographic images of the outer surface of the septum (**A**), the inner surface of the septum (**B**), and open mesh structure of the cell periphery (**C**) associated with the cell wall of *mecA*$^+$ *pbp2**. (i) Low-resolution AFM images and (ii) corresponding higher-resolution images of the region indicated by the white boxes in (i). Topographical height (z) range for (i) are 100 nm, 330 nm, and 85 nm and for (ii) are 11 nm, 16 nm, and 45 nm respectively. (**D-F**) AFM images of the same cell wall locations for *mecA*$^+$ *rpoB* pbp2**; (i) and (ii) as above. The height scales for (i) are 200 nm, 140 nm, and 130 nm. The height scales for (ii) are 16 nm, 9 nm, and 55 nm respectively. (**G-I**) AFM images of the PG structures associated with the following locations on the cell wall of *mecA*$^+$ *pbp2**; (**G**) AFM images of the outer surface of the septum, (**H**) inner surface of an incomplete septum, and (**I**) open mesh structure of the cell

periphery; (i) and (ii) as above. Topographical heights (z) for (i) are 150 nm, 210 nm, and 85 nm. Topographical heights (z) for (ii) are 11 nm, 22 nm, and 51 nm respectively. (**J-L**) AFM images of PG structures of the same locations in *mecA*$^{+}$ *rpoB** *pbp2**; (i) and (ii) as above. Topographical heights (z) for (i) are 140 nm, 200 nm, and 130 nm and for (ii) are 12 nm, 14 nm, and 70 nm respectively. (**M**), Plot of the measured thickness of dehydrated sacculi of *mecA*$^{+}$ *pbp2** and *mecA*$^{+}$ *rpoB** *pbp2** respectively. The number of independent fragments measured for each strain was 20. Data were analysed using the Mann-Whitney non-parametric statistical test (ns, not significant = 0.7180). (**N-O**) Cumulative fraction of total pore area as a function of the area of the pores distributed across the open mesh surface of the cell wall periphery surface (**N**) and the inner surface (**O**) of the septum of *mecA*$^{+}$ *pbp2** and *mecA*$^{+}$ *rpoB** *pbp2**. (**P**) Zoomed-in plot of the region highlighted by the dashed box in **(O)**. Data are representative of two independent biological repeats and five AFM independent images.

**Strain**	**Oxacillin MIC (µg ml^{-1})**	**Methicillin MIC (µg ml^{-1})**
SH1000 (SJF682)	≤ 0.25	0.5
mecA^{+} (SJF4996)	2	4
mecA^{+} *rpoB** (SJF5003)	> 256	> 256
COL (SJF315)	> 256	> 256
Mu50 (SJF5041)	> 256	> 256
MRSA252 (SJF4821)	> 256	> 256
TW20 (SJF6101)	> 256	> 256
USA300 (SJF4703)	0.75	1-2
USA300 (HL) (SJF6109)	> 256	64-128
EMRSA 15 (SJF6025)	16-24	64
EMRSA15 (HL) (SJF6110)	> 256	> 256
geh::mecA^{+} (SJF5324)	4-8	-
geh::mecA^{+} *rpoB** (SJF5323)	> 256	-
mecA^{+} *lytH* (SJF5461)	12	-
mecA^{+} *gdpP* (SJF5464)	≤ 2	-
mecA^{+} *pde2* (SJF5460)	3	-
mecA^{+} *rel** (SJF5463)	> 256	-
mecA^{+} *clpP* (SJF5459)	12-16	-
mecA^{+} *clpX* (SJF5462)	6-8	-
rpoB (*SJF5010)	≤ 0.25	-
lytH (SJF5455)	≤ 0.5	-
gdpP (SJF5025)	≤ 0.5	-
pde2 (SJF5454)	≤ 0.125	-
*rel** (SJF5457)	≤ 0.5	-
clpP (SJF5453)	0.25	-
clpX (SJF5456)	0.25-0.38	-

Table S1.

Oxacillin and Methicillin MICs for *S. aureus* strains. The MICs for oxacillin and methicillin were determined using E-test strips or the microdilution method (respectively) in triplicate as described in Materials and Methods. -, Not determined.

**Strain**	**Genotype and Markers**	**Source**
Escherichia coli		
NEB5α	*fhuA2 (argF-lacZ)U169 phoA glnV44 80 (lacZ)*M15 *gyrA96 recA1 relA1 endA1 thi-1 hsdR17*	New England Biolabs
Staphylococcus aureus		
SH1000	Functional *rsbU*$^+$ derivative of *S. aureus* 8325-4	*(50)*
COL	Healthcare acquired MRSA (HA-MRSA)	*(51)*
Mu50	HA-MRSA	*(19)*
MRSA252	HA-MRSA	*(18)*
TW20	HA-MRSA	*(17)*
USA300	Community-acquired MRSA (CA-MRSA)	*(20)*
USA300 (HL) (SJF6109)	High-level oxacillin resistant derivative of USA300	This study
EMRSA 15	CA-MRSA	*(3)*
EMRSA15 (HL) (SJF6110)	High-level oxacillin resistant derivative of EMRSA15	This study
VF17	SH1000 pGL485 (*lacI*); Cm^R	*(34)*
RN4220	Restriction deficient transformation recipient	*(52)*

CYL316	RN4220 pCL112Δ19; Cm^R	*(53)*
SJF4924	SH1000 *geh::Pspac-pbp2*, Kan^R	This study
SJF5046	SH1000 *lysA::mecA rpoB*$^{H929Q\textit{kan}}$; Ery^R, Kan^R	*(14)*
*pbp1** (SJF4656)	SH1000 *geh::Pspac-pbp1 pbp1::pbp1* lacI*; Tet^R, Cm^R	*(8)*
pbp1 mecA*$^+$ (SJF5223)	SH1000 *geh::Pspac-pbp1 pbp1::pbp1* lacI lysA::mecA*; Tet^R, Cm^R, Ery^R	*(8)*
pbp1 rpoB** (SJF5306)	SH1000 *geh::Pspac-pbp1 pbp1::pbp1* lacI rpoB*$^{H929Q\textit{kan}}$; Tet^R, Cm^R, Kan^R	This study
pbp1 mecA*$^+$ *rpoB** (SJF5226)	SH1000 *geh::Pspac-pbp1 pbp1::pbp1* lacI lysA::mecA rpoB*$^{H929Q\textit{kan}}$; Tet^R, Cm^R, Ery^R, Kan^R	*(8)*
Δ*pbp1* (SJF5106)	SH1000 *geh::Pspac-pbp1* Δ*pbp1 lacI*; Tet^R, Cm^R	*(8)*
Δ*pbp1 mecA*$^+$ (SJF5224)	SH1000 *geh::Pspac-pbp1* Δ*pbp1 lacI lysA::mecA*; Tet^R, Cm^R, Ery^R	*(8)*
Δ*pbp1 rpoB** (SJF5305)	SH1000 *geh::Pspac-pbp1* Δ*pbp1 lacI rpoB*$^{H929Q\textit{kan}}$; Tet^R, Cm^R, Kan^R	This study
Δ*pbp1 mecA*$^+$ *rpoB** (SJF5227)	SH1000 *geh::Pspac-pbp1* Δ*pbp1 lacI lysA::mecA rpoB*$^{H929Q\textit{kan}}$; Tet^R, Cm^R, Ery^R, Kan^R	*(8)*
pbp2 (SJF5630)	SH1000 *geh::Pspac-pbp2 pbp2::tet lacI*; Tet^R, Kan^R, Cm^R	This study
